# The chromosome-level genome assembly of *Aphidoletes aphidimyza* Rondani (Diptera: Cecidomyiidae)

**DOI:** 10.1038/s41597-024-03614-4

**Published:** 2024-07-17

**Authors:** Xiuxian Shen, Jianfeng Jin, Guoqiang Zhang, Bin Yan, Xiaofei Yu, Huizi Wu, Maofa Yang, Feng Zhang

**Affiliations:** 1 Institute of Entomology, Guizhou Provincial Key Laboratory for Agricultural Pest Management of the Mountainous Region, College of Agriculture, Guizhou University, Guiyang, 550025 China; 2 Department of Entomology, College of Plant Protection, Nanjing Agricultural University, Nanjing, 210095 China; 3 College of Tobacco Science, Guizhou University, Guiyang, 550025 China; 4 Zunyi Branch of Guizhou Tobacco Company, Zunyi, 564200 China

**Keywords:** Comparative genomics, Evolution

## Abstract

*Aphidoletes aphidimyza* is widely recognized as an effective predator of aphids in agricultural systems. However, there is limited understanding of its predation mechanisms. In this study, we generated a high-quality chromosome level of the *A. aphidimyza* genome by combining PacBio, Illumina, and Hi-C data. The genome has a size of 192.08 Mb, with a scaffold N50 size of 46.85 Mb, and 99.08% (190.35 Mb) of the assembly is located on four chromosomes. The BUSCO analysis of our assembly indicates a completeness of 97.8% (n = 1,367), including 1,307 (95.6%) single-copy BUSCOs and 30 (2.2%) duplicated BUSCOs. Additionally, we annotated a total of 13,073 protein-coding genes, 18.43% (35.40 Mb) repetitive elements, and 376 non-coding RNAs. Our study is the first time to report the chromosome-scale genome for the species of *A. aphidimyza*. It provides a valuable genomic resource for the molecular study of *A. aphidimyza*.

## Background & Summary

Aphids (Hemiptera: Aphididae) are prevalent insect pests that affect crops worldwide. They cause substantial economic losses by directly feeding on plants, spreading plant viruses, and producing honeydew^1–3^. Pesticides are mainly used to control aphids^4,5^. However, overuse of chemical pesticides can lead to drug resistance in aphids^6–8^ and may also kill various beneficial insects^9^. Therefore, alternative methods of pest control should be explored. The biological control method leverages living organisms to control pests and diseases. The use of natural enemies (such as birds, fungi, etc) to modulate the reproduction and transmission of pests^10–12^.

*Aphidoletes aphidimyza* Rondani (Diptera: Cecidomyiidae) is widely used to control aphids in agricultural systems^13^. It is an oligophagous insect that displays remarkable voracity and targets more than 80 species of aphids, including the major pests, namely *Aphis craccivora*^14^, *Aphis gossypii*^15^, *Myzus persicae*^16^, and others^17^. Owing to the limited dispersal ability of larvae, adults primarily depend on oviposition near aphid colonies to facilitate the predation of their progeny and the establishment of their population^13,18–20^. This is possibly based on chemosensory mechanisms, such as olfaction and gustation, which are also important for host selection. Olfaction is important for host orientation, while gustation is crucial in host selection^21–24^. Previous studies have shown that adults mainly rely on odor cues (such as aphid body volatiles, alarm pheromones, and aphid-induced plant volatiles) to precisely locate the position of aphids and complete oviposition^25–28^. They also use non-volatile cues, including honeydew, as a source of nutrition and an oviposition stimulant^29–31^. However, the lack of high-quality genomic data has limited the understanding of the genetic basis of search and predation on aphids.

In this study, we obtained a high-quality genome of Aphid midge using PacBio, Illumina, and Hi-C data. We annotated essential genomic elements, such as repeat elements, non-coding RNAs (ncRNAs), and protein-coding genes. The availability of a complete and detailed genome assembly is essential to basic biological research. This paper provides a valuable genomic resource for research into molecular mechanisms and evolution.

## Methods

### Sample collection

The larvae of *A. aphidimyza* were obtained from the tobacco base in Leshan Town, Zunyi City, Guizhou Province, China, in May 2017. They were raised in an artificial climate chamber (24 ± 1 °C with a 14:10 [L:D] h photoperiod, 70% relative humidity). In this experiment, initially, an inbred strain, a single pair of siblings was first used for 30 generations of mating, and then the genome and transcriptome of the inbred line were sequenced and analyzed. The larvae were fed with *Megoura japonica* on bean plants, and emerging adults were provided with 10% honey. A total of 500, 200, 200, and 200 female adult individuals were used for PacBio, Illumina, Hi-C, and Iso-Seq sequencing, respectively.

### Genome sequencing

Genomic DNA and RNA were extracted from the specimen using the FastPure® Blood/Cell/Tissue/Bacteria DNA Isolation Mini Kit (Vazyme Biotech Co., Ltd, Nanjing, China) and TRIzol reagent (YiFeiXue Tech, Nanjing, China), respectively. The quality and quantity of both total DNA and RNA were assessed through 1% agarose gel electrophoresis, the NanoDrop 2000 by Thermo Fisher, and the Qubit 3.0 fluorometer (Invitrogen, USA). PacBio library of a 30 kb insert size was created using the SMRTbell Template Prep Kit 2.0 from Pacific Biosciences of California, based in Menlo Park, USA. For Illumina sequencing, a short library with 150 bp paired-end reads and a 350 bp insert size was generated using the TruSeq DNA PCR-free kit. Furthermore, an Iso-Seq library with a 2 kb insert size was established using the SMRTbell prep kit 3.0 (Pacific Biosciences of California, Menlo Park, USA). Short RNA-seq libraries were also constructed for RNA sequencing on the BGIMGISEQ-500 platform (Shenzhen, China). The Hi-C sequencing was carried out by digesting extracted DNA with the Mbol restriction enzyme. We utilized the Illumina NovaSeq. 6000 platform to sequence all short-read libraries. PacBio sequencing was carried out using the PacBio Sequence RSII platforms employing the CLR mode. All these libraries were created and sequenced by Berry Genomics (Beijing, China). Our sequencing efforts yielded a total of 101.38 Gb of clean data, comprising 31.76 Gb from PacBio (168×), 26.64 Gb from Illumina (139×), 35.05 Gb from Hi-C (185×), and 7.93 Gb from RNA (6.28 Gb from Illumina and 1.65 Gb from Iso-Seq), as detailed in Table 1.

**Table 1:** Sequencing data was generated for the *A. aphidimyza* genome assembly and annotation.

Libraries	Insert sizes (bp)	Clean data (Gb)	Sequencing coverage (x)	SRA accession number
Illumina	300	26.64	139	SRR13333790
PacBio	30,000	31.76	168	SRR13222407
Hi-C	300	35.05	185	SRR13236663
Iso-Seq	5,000	1.65	—	SRR13333789
RNA	300	6.28	—	SRR13236725

### Genome survey and assembly

**Fig. 1:**
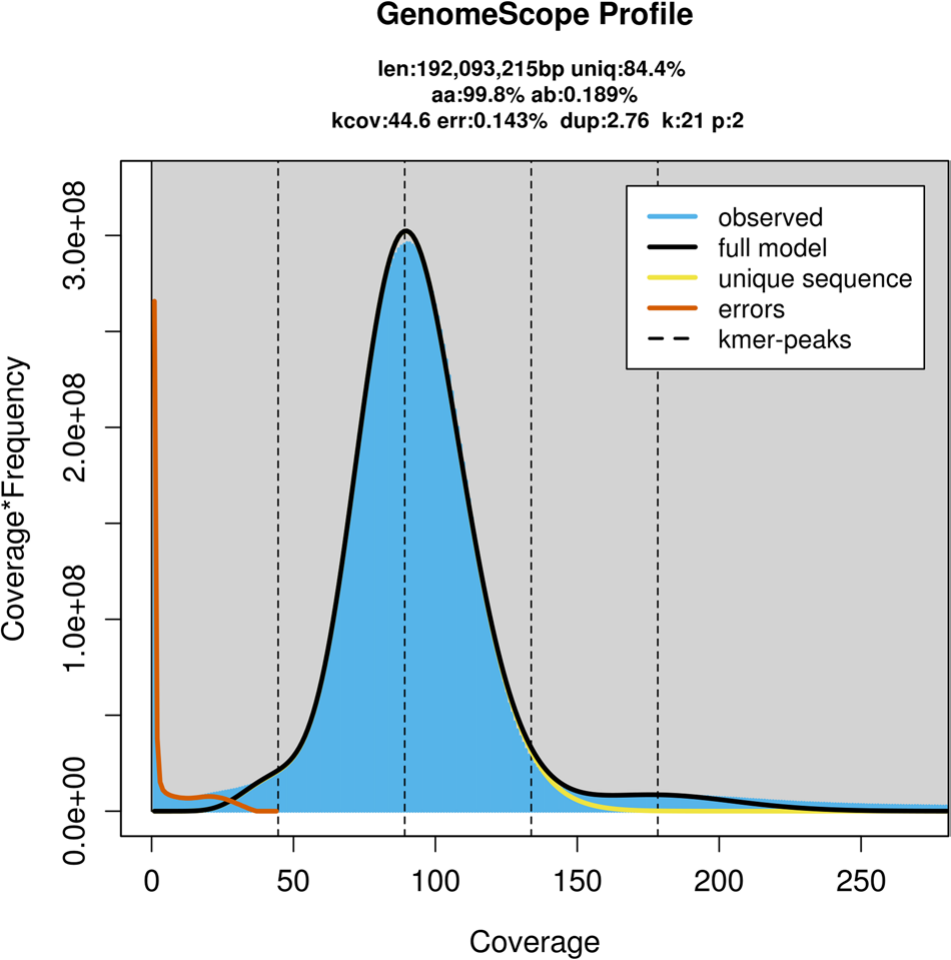
Genome survey at 21-mer of *A. aphidimyza* estimated by GenomeScope. The vertical dotted lines represent the peaks of different coverages for the heterozygous, the homozygous, and the duplicated sequences, separately.

We used BBTools v38.82^32^ to perform quality control on raw Illumina data, and then eliminated duplicate reads using “clumpify.sh”. Furthermore, “bbduk.sh” was used to trim sequences with quality scores below 20, sequences containing more than 5 Ns, and reads shorter than 15 bp. Polymer trimming (>10 bp) and correction of overlapping paired reads were also performed. In addition, a 21-mer was selected for k-mer analysis and the k-mer distribution was estimated using “khist.sh” (BBTools). The 21-mer depth frequency distribution was calculated using GenomeScope v2.0^33^ and the maximum k-mer coverage cut-off was set to 10,000. A k-mer analysis indicated that the number of unique k-mer spoke at 21 and predicted a genome assembly size of 192.09 Mb, with a heterozygosity of 0.189% and a repeat content proportion of approximately 15.4% (Fig. 1).

**Fig. 2:**
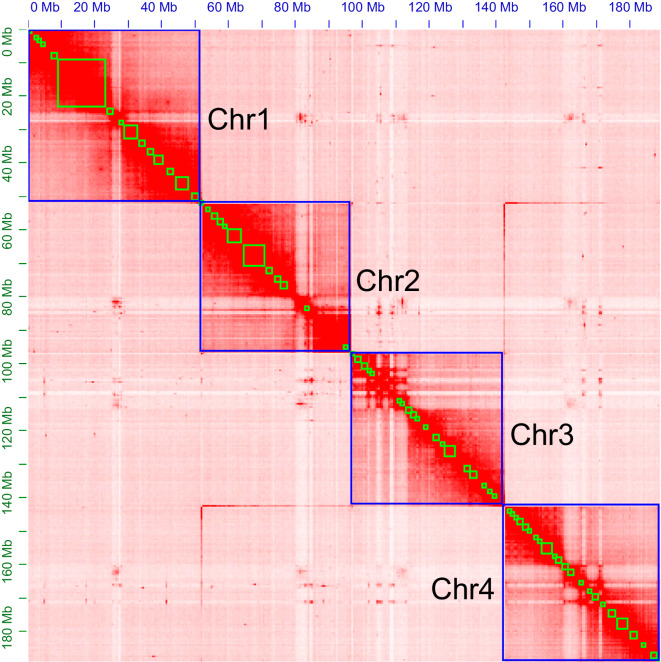
Genomic heatmap. Genome-scale chromosome heatmap of *A. aphidimyza*, with individual chromosomes outlined in blue.

**Fig. 3:**
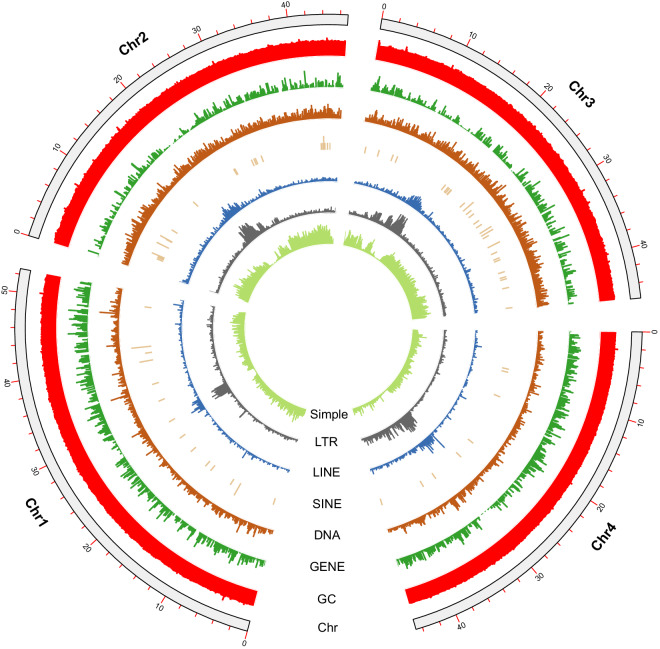
Genomic features. Circos plot with a window size of 100 kb. Each circle from inside to outside represents simple repeats, LTR, LINE, SINE, DNA, gene density, GC content, and chromosome length.

Primary assembly from PacBio reads was performed using Flye v2.8.3^34^, which involves one round of self-polishing with a minimum overlap of 3,000 (-i 1 -m 3000). The resulting assembly was polished with two rounds of short reads using NextPolish v1.3.1^35^. Heterozygous regions were eliminated using Purge_Dups v1.2.5^36^ with a 70% cut-off for identifying contigs as haplotigs. Minimap2 v2.23^37^ was used as the read mapper to remove redundancy and polish assembly. Hi-C reads were aligned to the assembly using Juicer v1.6.2^38^. Then, 3D-DNA v180922^39^ was used to anchor the contigs onto the chromosomes. Hi-C heatmaps were manually inspected and corrected using Juicebox v1.11.08^39^ to identify potential errors. Possibilities of contaminants were detected using MMseqs. 2 v11^40^, which performed Basic Local Alignment Search Tool (BLASTN)-like searches based on the NCBI nucleotide and UniVec databases with a sequence identity of 0.8 (“-min-seq-id 0.8”). To further examine vector contaminants, we used blastn (BLAST+ v2.11.0^41^) against the UniVec database. We considered that sequences with over 90% hits in the aforementioned database likely contained contaminants. Online BLASTN analysis in the NCBI nucleotide database was used to double-check sequences with above 80% hits. Following that, we removed any possible bacterial contamination from the assembled scaffolds. Our final genome assembly encompassed 192.08 Mb and comprised 70 scaffolds along with 444 contigs. It featured a scaffold N50 length of 46.85 Mb and a contig N50 size of 1.22 Mb (Fig. 2). The final assembly is close to the size of the genome survey (192.09 Mb) analysis. A remarkable 99.08% (190.35 Mb) of the genome was anchored into four chromosomes, as illustrated in Fig. 3 and detailed in Table 2. The assembled genome size closely resembled that of *Contarinia nasturtii*^42^(185.89 Mb).

**Table 2:** Genome assembly statistics for *A. aphidimyza*.

Assembly	Total length (Mb)	Number scaffolds (chromosomes)	Scaffold N50 length (Mb)	GC (%)	BUSCO (n = 1,367) (%)			
					C	D	F	M
Flye	197.50	711	1.22	35.02	97.8	2.6	0.4	1.8
Purge_Dups	192.77	467	1.22	35.01	97.7	2.2	0.4	1.9
NextPolish	192.68	467	1.22	35.01	97.7	2.2	0.4	1.9
3D-DNA	192.08	70 (4)	46.85	35.01	97.8	2.2	0.4	1.8
Final	192.08	70 (4)	46.85	35.01	97.8	2.2	0.4	1.8

Note: Complete BUSCOs (C); Complete and single-copy BUSCOs (S); Complete and duplicated BUSCOs (D); Fragmented BUSCOs (F); Missing BUSCOs (M)

### Genome annotation

The annotated *A. aphidimyza* genome included the following three important genomic components: repetitive elements, ncRNAs, and protein-coding genes. The *de novo* repeat library was established by RepeatModeler v2.0.3^43^ with the parameter “-LTRStruct”. We then combined Dfam 3.5^44^ and RepBase-20181026 databases^45^ to generate a custom library, which was employed to mask repeat elements using RepeatMasker v4.1.2-p1^46^. To summarize, RepeatMasker analysis revealed that the *A. aphidimyza* genome contains approximately 18.43% (35.40 Mb) repeat elements, i.e. long terminal repeat elements (LTR, 3.61%), DNA transposons (1.50%), long interspersed nuclear elements (LINE, 1.02%), and short interspersed nuclear elements (SINE, 0.01%), and other elements (Table 2). The annotations of rRNA, snRNA, and miRNA were compared with the Rfam v14.10 database using Infernal v1.1.4^47^ and tRNAscan-SE v2.0.9^48^. We identified 376 ncRNAs in the genome of *A. aphidimyza*, including 84 ribosomal RNAs, 52 miRNAs, 38 small nuclear RNAs, and 202 tRNAs (Table 3).

**Table 3:** Comparative statistics of *A. aphidimyza* and *Contarinia nasturtii* genome assembly and annotation.

	Characteristics	*A. aphidimyza*	*C. nasturtii*
Assembly	Genome size (Mb)	192.08	185.89
	Contig N50 (Mb)	1.21	0.095
	Scaffold N50 (Mb)	46.85	4.65
	Contigs number	444	8,114
	Scaffolds number	70	5,545
	Pseudochromosomes number	4	4
	GC%	35.01	33.76
	BUSCO completeness (%)	97.8	98.4
Annotation	No. of protein-coding genes	13,073	14,889
	Mean protein length (aa)	605.01	—
	BUSCO completeness (%)	98.6	92.9
	Repetitive elements Size (Mb)	35.40 (18.43%)	—
	DNA transposons (Mb)	2.85 (1.50%)	—
	SINEs (kb)	25.03 (0.01%)	—
	LINEs (Mb)	1.98 (1.02%)	—
	LTRs (Mb)	6.95 (3.61%)	—
	Unclassified (Mb)	15.78 (8.22%)	—
	No. of ncRNAs	376	—
	rRNA	84	—
	miRNA	52	—
	snRNA	38	—
	tRNA	202	—

 Protein-coding genes were annotated by combining results from *ab initio*, transcriptomic data, and protein homology using the MAKER pipeline v3.01.03^49^. BRAKER v2.1.6^50^ and GeMoMa v1.7.1^51^ predictions were combined as the *ab initio* input for MAKER, which combined transcriptome and protein evidence. Transcriptome data was used for annotation using a mixed assembly of Iso-seq and RNA-seq data. Transcriptome alignment was performed using HISAT2 v2.2.1^52^ and then assembled into transcripts using Stringtie v2.1.6^53^. BRAKER, employing Augustus v3.4.0^54^ and GeneMark-ES/ET/EP v4.68_lic^55^, was used to automatically train prediction models. This mode was based on RNA-seq alignments and reference proteins obtained from the OrthoDB10 v1 database^56^. GeMoMa predicted genes using protein homology, intron conservation, and transcripts. GeMoMa was used with the parameters “GeMoMa.c = 0.5 GeMoMa.p = 8” and protein sequences from five species, namely *Contarinia nasturtii*^42^ (GCF_009176525.2), *Bradysia coprophila*^57^ (GCF_014529535.1), *Anopheles arabiensis*^58^ (GCF_016920715.1), *Drosophila melanogaster*^59^ (GCF_000001215.4), and *Bombyx mori*^60^ (GCF_014905235.1). In addition, the protein sequences obtained from the same set of five species used in the GeMoMa analysis were included in the MAKER pipeline as supporting evidence for protein homology. To summarize, 13,073 protein-coding genes were annotated in *A. aphidimyza*. The number of PCGs in *A. aphidimyza* was fewer than that of *C. nasturtii* (14,889 genes) (Table 3). The completeness of 98.6% of *A. aphidimyza* was confirmed by Benchmarking Universal Single-Copy Orthologs (BUSCO), which was much higher than that of *C. nasturtii* (92.9%) (Table 3). Functional annotation of PCGs was performed using Diamond v2.0.8^61^, which used the sensitive mode and an e-value of 1e-5 to explore the UniProtKB database. Furthermore, we used EggNOG-mapper v2.1.5^62^ and InterProScan 5.48–83.0^63^ software to explore Gene Ontology (GO), enzyme codes (EC), Kyoto Encyclopedia of Genes and Genomes (KEGG) orthologous groups, clusters of orthologous groups (COG), and KEGG pathway annotations. Structural domains of genes were predicted using InterProScan, including the following five databases: Pfam^64^, Simple Modular Architecture Research Tool (SMART^65^), Superfamily^66^, Gene3D^67^, and Conserved Domain Database (CDD^68^). Finally, Genes with 9,798 GO terms, 8,646 KEGG pathways, 2,594 Enzyme Codes, 9,799 Reactome pathways, and 10,774 COG categories were identified by combining the eggNOG and InterProScan annotation results (Table 4).

**Table 4:** Functional annotation of the *A. aphidimyza* genome assembly.

Function annotation	Number
Number of genes matching Uniprot records	11,642
Number of genes labelled as “Uncharacterized protein”	906
Number of genes labelled as “unknown function”	1,503
Number of genes with InterProScan annotations	11,204
Number of genes with GO items from InterProScan annotations	7,401
Number of genes with KEGG pathway items from InterProScan annotations	7,533
Number of genes with MetaCyc items from InterProScan annotations	7,848
Number of genes with Reactome items from InterProScan annotations	9,799
Number of genes with gene names (function) from eggNOG annotations	2,819
Number of genes with Enzyme Codes (EC) from eggNOG annotations	2,594
Number of genes with COG Functional Categories from eggNOG annotations	10,774
Number of genes with GO items from eggNOG annotations	7,719
Number of genes with KEGG ko terms from eggNOG annotations	7,503
Number of genes with KEGG pathway terms from eggNOG annotations	4,311
Number of genes with GO items (combining InterProScan and eggNOG results)	9,798
Number of genes with KEGG pathways items (combining InterProScan and eggNOG results)	8,646

## Data Records

The raw sequencing data and genome assembly of *Aphidoletes aphidimyza* have been deposited at the National Center for Biotechnology Information (NCBI). The Illumina, Iso-Seq, Hi-C, PacBio, and RNA-seq data can be found under identification numbers SRR13333790^69^, SRR13333789^70^, SRR1323666380^71^, SRR13222407^72^, SRR13236725^73^, respectively. The assembled genome has been deposited in the NCBI assembly with the accession number GCA_030463065.1^74^. Additionally, the results of annotation for repeated sequences, gene structure, and functional prediction have been deposited in the figshare^75^.

## Technical Validation

Two independent methods were used to assess the completeness and quality of our genome assembly. We first used BUSCO v5.44^76^ with the “insecta_odb10” database (n = 1,367) to examine the completeness of the final assembled genome. In our BUSCO analysis, a commendable 97.8% of complete BUSCOs were identified, which included 95.6% of single-copy genes and 2.2% of duplicated BUSCOs (Table 2). To evaluate mapping success, we employed Minimap2 and SAMtools v1.9^77^ to align the clean reads obtained from both Illumina and PacBio sequencing with the final assembly. Impressively, we accomplished a mapping rate of 94.78% for Illumina reads, 98.09% for PacBio reads, 94.26% for Iso-seq reads, and 87.73% for RNA-seq reads, respectively. Overall, these assessments reflect the high quality of the genomic assembly.

## Data Availability

No specific script was used in this work. All commands and pipelines used in data processing were executed according to the manual and protocols of the corresponding bioinformatic software.
